# Gene regulation by the act of long non-coding RNA transcription

**DOI:** 10.1186/1741-7007-11-59

**Published:** 2013-05-30

**Authors:** Aleksandra E Kornienko, Philipp M Guenzl, Denise P Barlow, Florian M Pauler

**Affiliations:** 1 CeMM Research Center for Molecular Medicine of the Austrian Academy of Sciences, Lazarettgasse 14, AKH-BT25.3, 1090, Vienna, Austria

**Keywords:** Gene expression regulation, Histone modifications, lincRNA, lncRNA, Silencing, Transcriptional interference

## Abstract

Long non-protein-coding RNAs (lncRNAs) are proposed to be the largest transcript class in the mouse and human transcriptomes. Two important questions are whether all lncRNAs are functional and how they could exert a function. Several lncRNAs have been shown to function through their product, but this is not the only possible mode of action. In this review we focus on a role for the process of lncRNA transcription, independent of the lncRNA product, in regulating protein-coding-gene activity *in cis*. We discuss examples where lncRNA transcription leads to gene silencing or activation, and describe strategies to determine if the lncRNA product or its transcription causes the regulatory effect.

## LncRNAs - a new layer of genome regulatory information

It is now well appreciated that less than two percent of the human genome codes for proteins and the majority of the genome gives rise to non-protein-coding RNAs (ncRNAs) [1], which are predicted to play essential roles in a variety of biological processes [2,3].

The focus of this review is long ncRNAs (known as lncRNAs), which constitute the biggest class of ncRNAs with approximately 10,000 lncRNA genes so far annotated in humans [4]. lncRNAs are RNA polymerase II (RNAPII) transcripts that lack an open reading frame and are longer than 200 nucleotides. This size cut-off distinguishes lncRNAs from small RNAs such as microRNAs, piwi-interacting RNAs (piRNAs), small nucleolar RNAs (snoRNAs) and small interfering RNAs (siRNAs) and arises from RNA preparation methods that capture RNA molecules above this size. Although the function of most lncRNAs is unknown, the number of characterized lncRNAs is growing and many publications suggest they play roles in negatively or positively regulating gene expression in development, differentiation and human disease [2,5-10]. lncRNAs may regulate protein-coding (pc) gene expression at both the posttranscriptional and transcriptional level. Posttranscriptional regulation could occur by lncRNAs acting as competing endogenous RNAs to regulate microRNA levels as well as by modulating mRNA stability and translation by homologous base pairing, or as in the example of NEAT1 that is involved in nuclear retention of mRNAs [11]. In this review we focus on the regulation at the transcriptional level.

## Modes of transcriptional regulation by lncRNAs

Regulation of transcription is considered to be an interplay of tissue and developmental-specific transcription factors (TFs) and chromatin modifying factors acting on enhancer and promoter sequences to facilitate the assembly of the transcription machinery at gene promoters. With a growing number of lncRNAs implicated in transcriptional gene regulation, this view may need refinement to include networks of tissue and developmental-stage specific lncRNAs that complement known regulators to tightly control gene expression and thereby organism complexity [12,13]. Transcriptional regulation by lncRNAs could work either in *cis* or in *trans*, and could negatively or positively control pc gene expression. lncRNAs work in *cis* when their effects are restricted to the chromosome from which they are transcribed, and work in *trans* when they affect genes on other chromosomes.

## Regulation in *trans*

**Figure 1:**
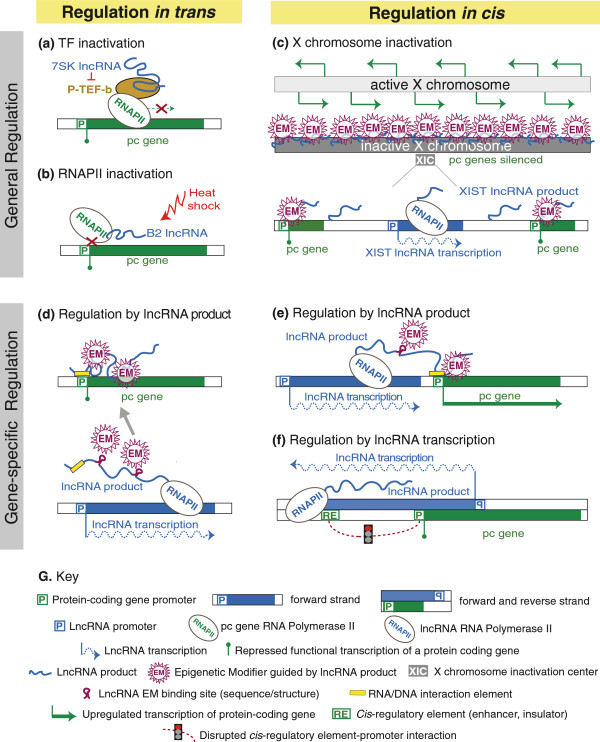
**Long non-protein-coding RNAs (lncRNAs) act at different levels to regulate protein coding gene expression.** lncRNAs can inhibit general protein-coding (pc) gene expression in *trans* (**a**) by preventing transcription factor (TF) activity (7SK lncRNA) or (**b**) by inhibiting RNAPII binding to DNA (B2 lncRNA). *Xist* lncRNA is transcribed from the X inactivation center (XIC) and inactivates a whole chromosome in *cis* (**c)** by recruiting epigenetic modifiers (EM). lncRNAs can regulate specific genes, acting in *trans* like *HOTAIR* (**d**) or in *cis* like *HOTTIP* (**e**) by directly recruiting epigenetic modifiers to certain genomic loci. In both cases the lncRNA binds EMs via a specific sequence or structure and targets them to promoter regions via DNA/RNA interaction elements to affect expression of the respective pc gene. Transcription of a lncRNA through a pc gene promoter or a *cis-*regulatory element (RE) affects pc gene expression in *cis* independent of the lncRNA product (**f**) by mechanisms discussed in the text. Both DNA strands are shown as separate boxes to indicate lncRNA transcription over the pc gene promoter in the antisense orientation. For details see text.

Some significant examples of lncRNAs that act in *trans* are those that can influence the general transcriptional output of a cell by directly affecting RNAPII activity (Figure 1a,b). One example is the 331 nucleotide 7SK lncRNA, which represses transcription elongation by preventing the PTEFβ transcription factor from phosphorylating the RNAPII carboxy-terminal domain (CTD) [14] (Figure 1a). Another example is the 178 nucleotide B2 lncRNA, a general repressor of RNAPII activity upon heat shock [15]. The B2 lncRNA acts by binding RNAPII and inhibiting phosphorylation of its CTD by TFIIH, thus disturbing the ability of RNAPII to bind DNA [16,17].

Regulation in *trans* can also act locus-specifically. While the ability of lncRNAs to act locus-specifically to regulate a set of genes was first demonstrated for imprinted genes where lncRNA expression was shown to silence from one to ten flanking genes in *cis*[18-20], lncRNAs that lie outside imprinted gene clusters, such as the *HOTAIR* lncRNA, were later found also to have locus-specific action. *HOTAIR* is expressed from the *HOXC* cluster and was shown to repress transcription in *trans* across 40 kb of the *HOXD* cluster [21]. *HOTAIR* interacts with Polycomb repressive complex 2 (PRC2) and is required for repressive histone H3 lysine-27 trimethylation (H3K27me3) of the *HOXD* cluster. Targeting of epigenetic modifiers (EMs) by lncRNAs provided a much sought after model to explain how EMs gain locus specificity (Figure 1d), and has since been suggested as a general mechanism for *trans*-acting lncRNAs [22,23].

## Regulation in *cis*

In contrast to *trans*-acting lncRNAs, which act via their RNA product, *cis*-acting lncRNAs have the possibility to act in two fundamentally different modes. The first mode depends on a lncRNA product. The major example of general *cis*-regulation is induction of X inactivation by the *Xist* lncRNA in female mammals. *Xist* is expressed from one of the two X chromosomes and induces silencing of the whole chromosome [24] (Figure 1c). As an example of locus-specific regulation it has been proposed that enhancer RNAs activate corresponding genes in *cis* via their product [25]. A well-studied *cis*-acting lncRNA acting through its product is the human HOTTIP lncRNA that is expressed in the HOXA cluster and activates transcription of flanking genes. HOTTIP was shown to act by binding WDR5 in the MLL histone modifier complex, thereby bringing histone H3 lysine-4 trimethylation (H3K4me3) to promoters of the flanking genes [26]. Such a mechanism in which a nascent lncRNA transcript binds and delivers epigenetic modifiers to its target genes while still attached to the elongating RNAPII is generally termed ‘tethering’ and is often used to explain *cis*-regulation by lncRNAs [23,27] (Figure 1e). It was also proposed to act in plants. In *Arabidopsis thaliana*, the *COLDAIR* lncRNA is initiated from an intron of the *FLC* pc gene and silences it by targeting repressive chromatin marks to the locus to control flowering time [28].

In contrast, the second mode of *cis* regulation by lncRNAs involves the process of transcription itself, which is *a priori cis*-acting (Figure 1f). Several lines of evidence suggest that the mere process of lncRNA transcription can affect gene expression if RNAPII traverses a regulatory element or changes general chromatin organization of the locus. In this review we discuss this underestimated role for lncRNA transcription in inducing protein-coding gene silencing or activation in *cis*, and overview possible mechanisms for this action in mammalian and non-mammalian organisms. Finally, we describe experimental strategies to distinguish lncRNAs acting as a transcript from those acting through transcription.

## Mechanisms by which lncRNA **transcription** silences gene expression

Transcription-mediated silencing, also referred to as ‘transcriptional interference’ (TI), is defined here as a case in which the act of transcription of one gene can repress in *cis* the functional transcription of another gene [29,30]. TI has been reported in unicellular and multicellular organisms [30]. Mechanistic details are still largely unclear, but TI could theoretically act at several stages in transcription: by influencing enhancer or promoter activity or by blocking RNAPII elongation, splicing or polyadenylation. All that would be required is that the RNA polymerase (RNAPII) initiated from an 'interfering' promoter traverses a 'sensitive' DNA regulatory sequence. TI has mainly been reported at overlapped promoters [31-35], but there are also examples where TI acts downstream of the promoter. In mouse, overlapping transcription controls polyadenylation choice of two imprinted genes [36,37]. In *Saccharomyces cerevisiae*, collisions between elongating antisense RNAPIIs can lead to stalling of both polymerases that is resolved by ubiquitylation-directed proteolysis, and this has been proposed to be a regulatory mechanism [38]. However, it is unknown if RNAPII collisions occur sufficiently frequently *in vivo* in yeast or other organisms to offer a means of regulating convergent genes, or if this mechanism could lead to an interfering RNAPII eliminating its sensitive collision partner. Despite these examples, the most common reports of TI concern an overlapped promoter, and in the following sections we describe studies investigating the molecular mechanisms underlying interference at the promoter.

## Transcriptional interference acting by promoter nucleosome repositioning

**Figure 2:**
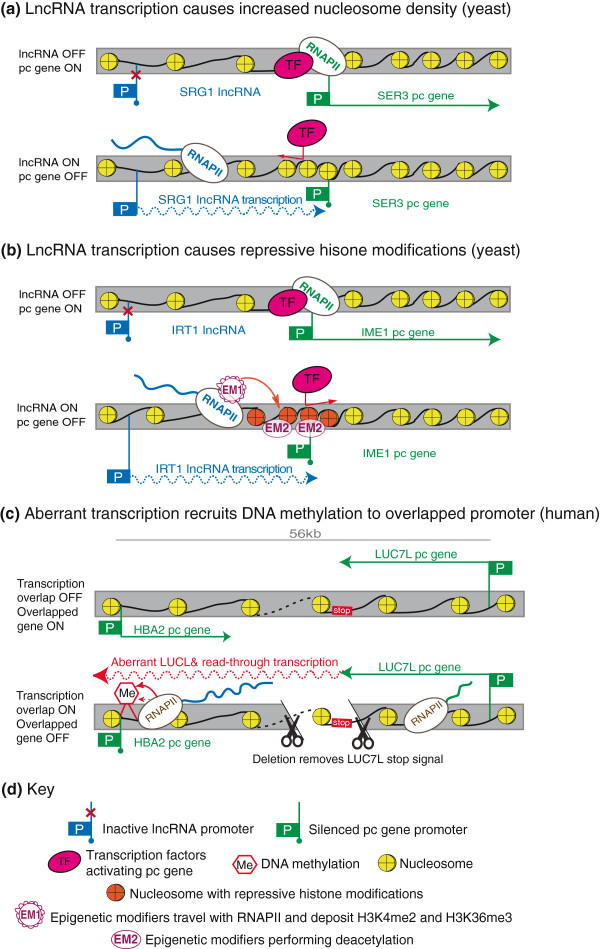
**Transcription interference-mediated silencing by chromatin changes.** (**a**) Top: in yeast the absence of *SRG1* lncRNA allows transcription machinery assembly at the *SER3* protein coding gene promoter. Bottom: *SRG1* lncRNA transcription causes dense nucleosome packing over the downstream *SER3* pc gene promoter that blocks TF binding and pc gene expression. (**b**) Top: in yeast the absence of *IRT1* lncRNA allows *IME1* pc gene expression. Bottom: RNAPII transcribing the *IRT1* lncRNA carries EMs that deposit repressive histone modifications at the *IME1* promoter (EM1 - methyltransferases). These modifications allow the binding of other EMs that remove active histone modifications (EM2 - deacetylases) and cause a repressive chromatin environment that blocks TF binding leading to silencing. (**c**) Top: in a healthy human, *LUC7L* and *HBA2* pc genes do not overlap and are both expressed. Bottom: a chromosomal deletion of the *LUC7L* transcriptional stop signal (red ‘stop’ box) causes transcription of the *LUC7L* pc gene through the promoter of the *HBA2* pc gene. By an unknown mechanism this aberrant transcription causes DNA methylation and silencing of the *HBA2* promoter. For details see Figure 1g and text.

DNA in the nucleus is organized into chromatin with the organizational scaffold consisting of nucleosomes, each with two copies of H3, H4, H2A and H2B histones [39]. Nucleosomes can be densely packed, interfering with protein-DNA interactions, or relaxed, facilitating these interactions [40]. The transcription process, which generates single-stranded DNA as RNAPII progresses along a gene locus, can directly affect nucleosome positioning [41-43] (reviewed in [44,45]). Thus, lncRNA transcription could cause TI by depositing nucleosomes in a manner unfavorable for TF binding on promoters or enhancers. An example of this mechanism is the silencing of the yeast *SER3* pc gene by transcriptional overlap by the *SRG1* lncRNA (Figure 2a) [46]. *SRG1* transcription increases nucleosome density at the overlapped *SER3* promoter. Deletion of three transcription elongation factors that are associated with the elongating polymerase and are necessary for nucleosome repositioning (*SPT16*, *SPT6*, *SPT2*) [47-49] abolished the silencing effect without stopping transcription of the overlapping lncRNA *SRG1*[50,51], indicating the necessity of chromatin reorganization for silencing. In contrast, deletion of epigenetic modifiers (such as SET1/2 histone methyltransferases and SET3C/RPD3S deacetylases described later) did not affect silencing, showing that nucleosome positioning, but not changes in histone modifications, is responsible for repression. The experiments did not directly exclude a role for the *SRG1* lncRNA product, but the silencing can be explained solely by the process of transcription [44,45]. TI by nucleosome repositioning may be a general mechanism in yeast, as the RNAPII elongation and chromatin organization factors responsible for *SER3* silencing are also known to be involved in the suppression of transcription initiation from cryptic promoters within the body of actively transcribed genes [52,53]. Since genes controlling RNAPII elongation and chromatin organization are largely conserved, it is possible that lncRNAs could use similar nucleosome repositioning silencing in mammals. This is supported by the example that chromatin reassembly factors are necessary for silencing an HIV provirus when integrated into an actively transcribed host gene in a human cell system [54].

## Transcriptional interference acting by promoter histone modifications

Promoter associated nucleosomes carry post-translational histone tail modifications that reflect the activity state of the promoter and also influence accessibility of DNA binding factors involved in transcription [55]. Active gene promoters correlate with H3 and H4 acetylation and with H3K4me3, while inactive promoters do not and, in mammals, they also gain repressive histone marks such as H3K9me3 or H3K27me3. Some histone modifying enzymes have been shown to bind and travel with elongating RNAPII [56,57], so it is possible that lncRNA transcription can induce TI by affecting histone modifications at the promoter of an overlapped target gene. For example, in yeast the SET1/2 methyltransferases, which induce H3K4me2 and H3K36me3 in the body of transcribed genes, bind and travel with elongating RNAPII [58-60]. These modifications in turn recruit the SET3C/RPD3S histone deacetylase complexes to create a chromatin environment repressive for transcription initiation [61-63].

Two studies indicate that this is a mechanism used by lncRNAs to induce TI in yeast. In the first study the *IME1* pc gene, which induces gametogenesis in diploid *S. cerevisiae* cells but is repressed in haploid cells, was shown to be silenced by the *IRT1* lncRNA that overlaps its promoter [64]. Genetic experiments repositioning the *IRT1* lncRNA distant from *IME1* on the same chromosome showed that *IRT1* transcriptional overlap of the *IME1* promoter is necessary for silencing. Interestingly, the instability of the *IRT1* lncRNA product and its non-specific cellular localization indicated the lncRNA product is unlikely to play a role in the silencing mechanism. Instead, *IRT1* lncRNA transcription through the *IME1* promoter reduced recruitment of the essential POG1 transcription factor, increased nucleosome density and induced the SET1/2 mediated cascades of histone modifications, which were shown to be necessary for silencing [64] (Figure 2b). In the second study lncRNA transcription was shown to be causative for silencing of the *GAL1* and *GAL10* genes, involved in galactose metabolism in *S. cerevisiae*. *GAL10* and *GAL1* are divergently transcribed from a bidirectional promoter. The 4 kb lncRNA, called *GAL10*-ncRNA, initiates in the body of the *GAL10* gene, and is transcribed through the *GAL10/GAL1* promoter antisense to the *GAL10* gene. *GAL10*-ncRNA transcription induces SET2-mediated establishment of H3K36me3 along its gene body, thereby recruiting RPD3S-dependent deacetylation that resulted in reduced transcription factor binding and repression of the *GAL1/GAL10* promoter [65]. Both SET3C and RPD3S are proposed to have a general role in repressing cryptic promoters within gene bodies [61,66] and a genome-wide study implied a role for SET3C in overlapping lncRNA-mediated silencing of a set of pc genes in yeast [66]. This indicates that the mechanism described above might be widely used to control gene expression in yeast. Although similar studies have not been described for the mammalian genome, H3K36me3 marks the body of transcribed genes in mammals, raising the possibility that such TI mechanisms could be conserved [56,57].

## Transcriptional interference acting by promoter DNA methylation

In mammalian genomes DNA methylation is generally associated with silent CpG island promoters, but the majority of CpG island promoters remain methylation free independent of their expression status [67-69]. The process of *de novo* methylation depends on the DNMT3A/3B methyltransferases and the catalytically inactive DNMT3L homologue and requires histones lacking H3K4me3, ensuring that active promoters remain methylation-free [70]. Notably, while DNA methylation at the promoter blocks transcription initiation, methylation in the gene body does not. Two important examples in humans based on genetic analyses indicate that DNA methylation can be involved in TI-induced silencing, although the causality between DNA methylation and silencing is still a matter of discussion [67]. One study of a patient with inherited α-thalassemia identified a deletion of the *LUC7L* 3' end that allowed aberrant transcription of *LUC7L* through the downstream *HBA2* gene, causing its silencing and the disease phenotype [71] (Figure 2c). Mouse models that mimicked the deleted genomic locus showed that the main cause of silencing was the acquisition of DNA methylation at the *HBA2* promoter. Notably, DNA methylation acquisition was not simply the consequence of an inactive promoter, as removal of *HBA2* transcription by deleting its TATA box did not induce methylation. The sequence of the *LUC7L* gene and thus the aberrant RNA product was also not essential for *HBA2* silencing, as replacing the *LUC7L* gene body with another protein-coding gene did not remove the repressive effect. In a second example, a subset of Lynch syndrome patients display DNA methylation and inactivation of the mismatch repair *MSH2* gene that correlates with aberrant transcription from the flanking *EPCAM* gene that carries a 3' deletion [72].

In both these examples, the molecular details of methylation establishment and the mechanism by which the methylation machinery targets the overlapped promoter are yet unknown. However, the data so far show that it is a *cis*-acting mechanism as only the allele carrying the deletion silences the overlapped protein-coding gene. In addition, although a role for the aberrant RNA product was not excluded, it appears unlikely that mutation-induced transcription of two independent intergenic chromosomal regions in the described diseases produces lncRNA products with similar repressive functions. Interestingly, the silencing of imprinted pc genes by lncRNAs is also often correlated with the gain of DNA methylation on the silent pc gene promoter [73]. In the case of the *Igf2r* gene, this DNA methylation mark is not necessary for initiation or maintenance of the silent state but seems to play a role in re-enforcing the silent state [35,74].

## Transcriptional interference in the absence of chromatin changes at the silenced promoter

**Figure 3:**
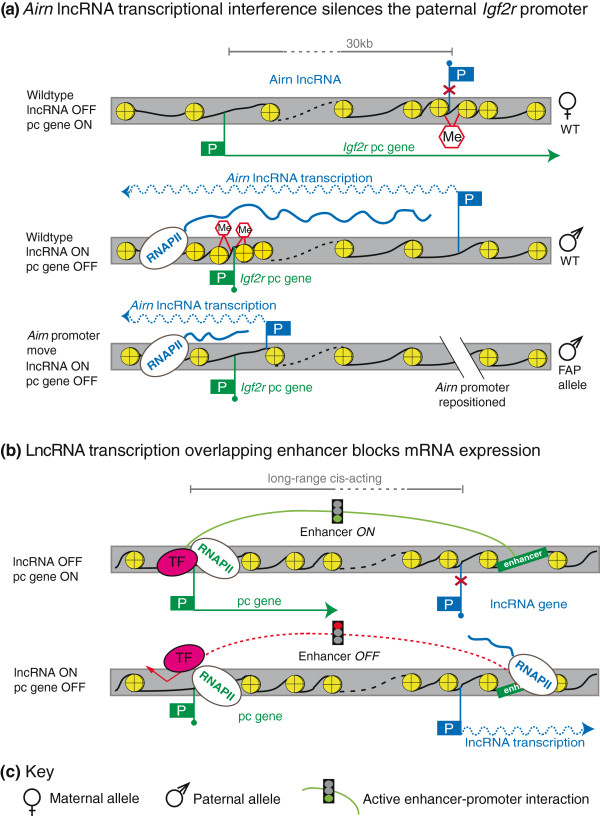
**Transcription interference-mediated silencing without chromatin changes.** (**a**) Top: a wild-type maternal allele does not express *Airn* lncRNA as its promoter is repressed by a DNA methylation imprint, thus allowing the *Igf2r* gene to be active. Middle: on the wild-type paternal allele *Airn* transcription overlaps with and silences the *Igf2r* pc gene promoter, independent of the *Airn* lncRNA product. The silent *Igf2r* promoter is marked by increased nucleosome density and DNA methylation in the absence of active histone modifications. Bottom: increased nucleosome density, loss of active histone marks and DNA methylation are not necessary for *Igf2r* repression as demonstrated by the FAP allele that moved the *Airn* promoter close to the *Igf2r* promoter and silenced *Igf2r* in the absence of repressive chromatin features. (**b**) Top: a hypothetical enhancer activates a pc gene by direct long-range DNA interactions. Bottom: transcription of a lncRNA overlapping the enhancer interferes with the DNA interaction and thereby silences the pc gene. For details see Figure 1g, Figure 2d and text.

In addition to RNAPII acting as a carrier of chromatin modifying enzymes, other TI models predict that RNAPII from one promoter traversing across another promoter can interfere with its activity without introducing chromatin changes [30,75,76]. An indication that such a mechanism can be used by lncRNAs in mammals comes from a study that used a genetic approach to dissect the silencing function of the imprinted mouse *Airn* lncRNA [77,78]. *Airn* is an inefficiently spliced 118 kb lncRNA expressed on paternally inherited chromosomes that overlaps and silences the promoter of the *Igf2r* pc gene - a dose-sensitive and essential embryonic growth suppressor [18,79] (Figure 3a). To determine if *Airn* transcription or its lncRNA product were required for silencing, homologous recombination in embryonic stem cells was used to shorten the length of *Airn*, either before or after the *Igf2r* promoter, by insertion of a polyadenylation cassette [35]. Notably, only shortened *Airn* variants that traversed the *Igf2r* promoter induced silencing. Furthermore, while *Igf2r* silencing is normally accompanied by DNA methylation, repressive histone marks and chromatin compaction of the silent *Igf2r* promoter [80,81], *Igf2r* silencing was not dependent on DNA methylation - in contrast to the silencing of *HBA2* by aberrant *LUC7L* transcription described above. Instead, *Airn* transcriptional overlap interfered with the accumulation of functional RNAPII on the *Igf2r* promoter in the presence of open chromatin [35]. Additional support for *Igf2r* silencing by *Airn* transcriptional interference is provided by genetic experiments that used an inducible *Airn* promoter to silence *Igf2r* at different stages of embryonic stem cell differentiation [74]. The demonstration that *Airn* transcription is continuously required for *Igf2r* silencing and that its silencing efficiency decreases when the *Igf2r* promoter is strongly expressed provides support for a model whereby RNAPII initiated from an 'interfering' promoter interferes with transcription initiation from a 'sensitive' promoter.

To date, other examples of lncRNAs acting by this mechanism in mammals are lacking. It has been suggested that silencing of an alternative promoter of the mouse *fpgs* pc gene is an example of transcription inducing silencing without introducing chromatin changes [82], but this system has not been subject to a similar genetic analysis and alternative explanations remain possible. How RNAPII from an interfering promoter is able to suppress functional transcription of the overlapped promoter remains to be determined, but stalling of the interfering RNAPII elongating over the sensitive promoter has been suggested to block access of essential TFs [30,83]. This mechanism should not be confused with the phenomenon of genome-wide RNAPII pausing at promoters, which represents an intermediate step between RNAPII initiation and elongation phases and might be a common mechanism regulating differential gene expression in metazoans [84,85].

The above examples describe repressive effects from RNAPII transcribing lncRNAs through promoters of silenced genes. However, transcriptional interference might also disrupt enhancer function when RNAPII traverses an enhancer, and this is an attractive model to explain the repression of a cluster of genes by a lncRNA in a tissue-specific manner [75] (Figure 3b). This situation arises in two imprinted gene clusters where the *Airn* and *Kcnq1ot1* lncRNAs each overlap one gene, but silence multiple genes in *cis* in a tissue-specific manner. The repressive histone EHMT2 methyltransferase has been shown to be necessary in the placenta to silence one of the three genes controlled by *Airn*[86]. The *Kcnq1ot1* lncRNA has been shown to silence multiple genes in placental cells by the action of repressive POLYCOMB histone modifying enzymes [87,88]. In both cases, a direct role for the lncRNA in targeting the histone modifying complexes was proposed, based on the findings that the lncRNAs interact with the respective histone modifying complex. This correlation-based evidence is, however, not sufficient to rule out the possibility that both lncRNAs silence distant genes by transcription alone (reviewed in [75,76]). In support of a transcription-based model, it was shown that *Kcnq1ot1* silences at least one gene by regulating chromatin flexibility and access to enhancers [89]. This is consistent with a two-step model whereby lncRNA transcription initiates silencing of non-overlapped genes by enhancer interference, then repressive histone modifying enzymes maintain that silencing.

## lncRNA transcription creating a permissive chromatin environment

**Figure 4:**
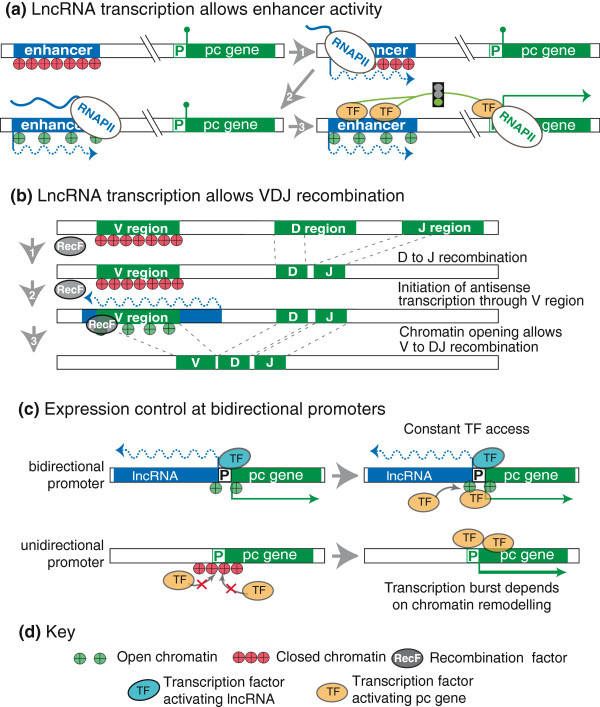
**Transcription of lncRNA creates permissive chromatin environment.** (**a**) Top left: an inactive enhancer with closed chromatin cannot activate the pc gene. Top right, bottom left: transcription of the enhancer opens chromatin. Bottom right: open chromatin at the enhancer allows TF binding and interaction with and activation of the pc gene promoter. (**b**) VDJ recombination. From top to bottom: 1, D and J segments are joined and the V region has closed chromatin; 2, antisense transcription through the V region opens the chromatin and allows recombination factors to bind; 3, a V segment is joined to the DJ segment. (**c**) Top: at a bidirectional promoter a lncRNA and a pc gene are transcribed in opposite directions. The promoter is always in an open chromatin conformation as either the lncRNA or the pc gene is transcribed, which is thought to reduce transcriptional noise. Bottom: a unidirectional pc gene promoter can acquire a closed chromatin conformation due to stochastic TF binding, which is thought to increase transcriptional noise. Noise defines the variation of expression of a transcript between genetically identical cells caused by the stochastic binding of TFs regulated by the local chromatin environment. For details see Figure 1g, Figure 2d, Figure 3c and text.

Enhancers are genetic elements that bind transcription factors facilitating transcription machinery assembly at nearby promoters [90,91]. RNAPII transcripts up to 2 kb long are transcribed bi-directionally from some neuronal enhancers (termed enhancer or eRNAs) [91,92]. Transcription of eRNAs positively correlated with expression of nearby mRNAs and a model was proposed, but not yet experimentally tested, in which their transcription establishes a chromatin landscape that supports enhancer function (Figure 4a). lncRNA transcription, either by opening chromatin or inhibiting repressor protein binding, could similarly result in gene or locus activation. One example of this is the process of V(D)J recombination, which joins elements of the V, D and J multigene family by chromosomal rearrangements to create functional B cell immunoglobulins and T cell receptors [93] (Figure 4b). The V, D and J genes lie next to each other on the same chromosome and antisense intergenic transcription through these genes is detected prior to the recombination process [94]. Genetic experiments have shown that intergenic lncRNA transcription is required for both B and T cell V(D)J recombination [95,96]. Similar correlations between intergenic transcription and gene expression were observed for the mouse *β-globin* locus [97] where promoter deletion experiments showed that lncRNA transcription was responsible for stable, active and hyper-accessible chromatin [98].

## lncRNA transcription and locus activation

Other examples indicate that lncRNA transcription activates gene expression by blocking access of repressor complexes to chromatin. In *Drosophila*, intergenic non-coding transcription at the *BITHORAX* complex (*BX-C*) is implicated in reversing *POLYCOMB* group (*PCG*)-mediated gene silencing and is correlated with an active chromatin state [99]. This mode of action was later suggested to be a general mechanism where the act of transcription serves as an epigenetic switch that relieves *PCG*-mediated gene silencing by recruiting epigenetic modifiers to induce gene expression and generate stable and heritable active chromatin [100]. In line with this hypothesis, intergenic transcription through *PCG* response elements (PREs) in the *BX-C* cluster is not only found during embryogenesis but also in late stage larvae, indicating that continuous transcription is required to keep genes active [101]. In mouse and human, a similar role for PRE transcription has been proposed. An analysis of lncRNA transcription in the human *HOXA* cluster revealed a positive correlation between lncRNA transcription and the loss of PCG/chromatin interactions that precedes *HOXA* gene activation [102]. Additionally, lncRNAs have been identified at promoter regions of PCG-regulated genes in mouse cells; while their role is not yet clear, it has been suggested that they either promote or interfere with PCG binding at target genes [103,104].

A further example of a lncRNA mediating chromatin opening was described at the *S. cerevisiae PHO5* gene. Transcription of an antisense lncRNA that initiates near the 3’end of *PHO5* and overlaps its gene body and promoter is associated with rapid activation of *PHO5* by enabling nucleosome eviction. Biochemical inhibition of RNAPII elongation as well as genetic disruption of lncRNA elongation demonstrated a direct role in *PHO5* activation [105]. The association of lncRNA transcription with gene activation needs, however, to be considered within the framework that most protein-coding gene promoters in yeast and mammalian cells give rise to a bidirectional antisense lncRNA transcript [106,107]. To date it is unclear if promoter-associated bidirectional lncRNAs represent spurious transcription in the context of open chromatin [108,109] or is required to maintain open chromatin. In the latter case enhanced TF binding ensures accessible chromatin that allows more constant pc gene expression within a cell population [110] (Figure 4c).

## Strategies for distinguishing a role for the lncRNA product from that of its transcription

Following genome-wide lncRNA mapping, functional studies so far have mainly focused on lncRNA products [7,111]. As it becomes clear that lncRNAs can act through their transcription, it is important to identify strategies to determine the function and mode of action of each particular lncRNA. One common starting point to determine lncRNA function has been RNA interference (RNAi)-mediated knockdown, despite long-standing observations that the RNAi machinery in mammalian cells is located in the cytoplasm [112]. While there is evidence that some RNA-induced silencing complex (RISC) components are found in the nucleus, functional complexes are specifically loaded in the cytoplasm, prohibiting the application of RNAi strategies for nuclear localized lncRNAs [113]. In contrast, antisense oligonucleotides (ASO) that work via an RNaseH-dependent pathway will deplete nuclear-localized lncRNAs [114,115]. However, three additional points of caution should be noted. First, non-specific effects arising from nuclear transfection reagents [116] have confused some observations. One critical validation step for knockdown studies would be a rescue experiment in which the lncRNA, modified to be invulnerable to the knockdown, is expressed as a transgene under the same transfection conditions [111]. Second, some results have highlighted major differences when functional studies used post-transcriptional depletion strategies in cell lines in contrast to genetic studies in the organism. Notable examples are *Neat1*[117], *Malat1*[116,118,119] and *Hotair*[120] where studies of mice carrying genetically disrupted alleles of these three lncRNAs failed to reproduce phenotypes deduced from cell lines following RNAi, ASO or over-expression studies. Third, while knockdown experiments may elucidate the function of lncRNAs acting through their product, the function of *cis*-acting lncRNAs that depend only on transcription will not be disturbed.

Features such as subcellular localization, half-life and steady-state abundance would form a good basis to allow functional tests to be designed. In addition, knowledge of the lncRNA splicing efficiency, conservation of splicing pattern in multiple tissues and species, an estimation of transcript repeat content and, finally, an accurate mapping of lncRNA 5' and 3' ends are essential preliminary steps. We have previously proposed that a subclass of lncRNAs, ‘macro’ lncRNAs, show RNA biology hallmarks such as inefficient splicing, extreme length, high repeat content, lack of conservation and a short half-life. These features are also indicators that the lncRNA product is less important than the act of transcription [121]. Once RNA biology features are known, experiments can be designed to distinguish between a role for the lncRNA product or its transcription.

From the caveats of posttranscriptional knockdown experiments described above, it becomes clear that genetic strategies are optimal for testing lncRNA function. These strategies include manipulating the endogenous locus to delete the promoter or the whole gene or to shorten its length using inserted polyadenylation signals, as described for several examples above. This may appear a formidable task with the appreciation that lncRNAs in the human genome may outnumber protein-coding genes [4]; however, suitable cell systems already exist. These include the use of haploid cell lines with transcriptional stop signal insertions in most human genes that are screened by RNA sequencing [122], gene targeting by engineered zinc-finger nucleases [123] or CRISPR systems [124] or the use of mouse embryonic stem cells that have efficient rates of homologous targeting [125,126].

**Figure 5:**
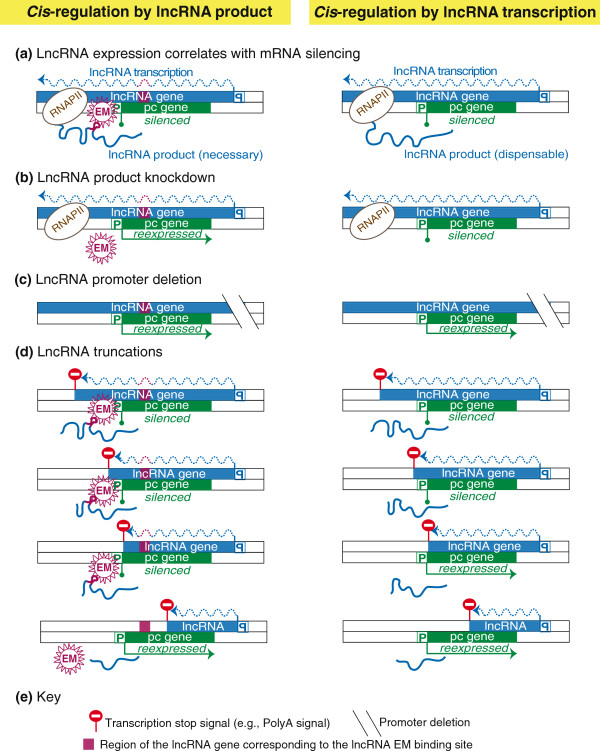
**Strategies to distinguish between the function of a lncRNA product and its transcription.** Both DNA strands are shown as separate boxes to indicate a lncRNA transcribed from the top reverse strand, overlapping a pc gene transcribed from the bottom forward strand in antisense orientation. A silencing function of the lncRNA can be predicted by an anti-correlating expression pattern. (**a**) Left: the lncRNA silencing effect is mediated by tethering of the lncRNA product at the site of transcription, sequence-specific binding of an EM to the lncRNA and guidance of the EM to the pc gene promoter. Right: silencing is mediated by a transcription process independent of the lncRNA product. (**b**) Posttranscriptional knockdown removes the lncRNA product, thus reversing a lncRNA product-mediated effect (left) but not the transcription-mediated effect (right). (**c**) lncRNA promoter deletion removes both lncRNA product- (left) and transcription-mediated (right) effects. (**d**) Truncation experiments inserting transcriptional stop signals at different positions within the lncRNA gene identify the functional region of the lncRNA gene (RNAPII is not shown). Left: lncRNA is only functional when the region corresponding to EM binding site is present. Right: lncRNA is only functional when it crosses the promoter of the overlapped pc gene. For details see Figure 1g, Figure 2e, Figure 3c, Figure 4d and text.

These genetic strategies could be applied to determine if the lncRNA is functional and if its function requires the lncRNA product or only depends on the act of transcription (Figure 5). Once these answers are obtained, it will be useful to test whether additional chromatin features are involved. This could include chromatin accessibility assays to address nucleosome density in the regulated gene; and mapping of histone modifications and DNA methylation, and of the presence of RNAPII and other transcription machinery components. These studies have been made easier in the mouse and human genome due to the publicly available ENCODE data [127]. As lncRNA identification becomes easier due to improved sequencing and bioinformatics tools, the number of annotated lncRNA transcripts is rising sharply [4,128]. It is therefore a high priority to determine which lncRNAs are functional and which represent spurious transcription [109,129]. To date only a relatively small number of mammalian lncRNAs have clearly been shown to regulate gene expression and most attention has centered on lncRNAs that act through their transcription product [23]. With the recent demonstration that for some mammalian lncRNAs the act of their transcription is sufficient for function [35], it becomes clear that there can be a number of lncRNAs acting in a similar way. If the above described findings and approaches are used as guidelines, many new lncRNAs regulating genes by the act of transcription are likely to be discovered.

## References

[B1] Djebali S Davis CA Merkel A Dobin A Lassmann T Mortazavi A Tanzer A Lagarde J Lin W Schlesinger F Xue C Marinov GK Khatun J Williams BA Zaleski C Rozowsky J Röder M Kokocinski F Abdelhamid RF Alioto T Antoshechkin I Baer MT Bar NS Batut P Bell K Bell I Chakrabortty S Chen X Chrast J Curado J Landscape of transcription in human cells Nature 2012 489 101 108 22955620 10.1038/nature11233 PMC3684276

[B2] Wilusz JE Sunwoo H Spector DL Long noncoding RNAs: functional surprises from the RNA world Genes Dev 2009 23 1494 1504 19571179 10.1101/gad.1800909 PMC3152381

[B3] Pauli A Rinn JL Schier AF Non-coding RNAs as regulators of embryogenesis Nat Rev Genet 2011 12 136 149 21245830 10.1038/nrg2904 PMC4081495

[B4] Derrien T Johnson R Bussotti G Tanzer A Djebali S Tilgner H Guernec G Martin D Merkel A Knowles DG Lagarde J Veeravalli L Ruan X Ruan Y Lassmann T Carninci P Brown JB Lipovich L Gonzalez JM Thomas M Davis CA Shiekhattar R Gingeras TR Hubbard TJ Notredame C Harrow J Guigó R The GENCODE v7 catalog of human long noncoding RNAs: analysis of their gene structure, evolution, and expression Genome Res 2012 22 1775 1789 22955988 10.1101/gr.132159.111 PMC3431493

[B5] Taft RJ Pang KC Mercer TR Dinger M Mattick JS Non-coding RNAs: regulators of disease J Pathol 2010 220 126 139 19882673 10.1002/path.2638

[B6] Huarte M Rinn JL Large non-coding RNAs: missing links in cancer? Hum Mol Genet 2010 19 R152 R161 20729297 10.1093/hmg/ddq353 PMC2953740

[B7] Guttman M Donaghey J Carey BW Garber M Grenier JK Munson G Young G Lucas AB Ach R Bruhn L Yang X Amit I Meissner A Regev A Rinn JL Root DE Lander ES: lincRNAs act in the circuitry controlling pluripotency and differentiation Nature 2011 477 295 300 21874018 10.1038/nature10398 PMC3175327

[B8] Gupta RA Shah N Wang KC Kim J Horlings HM Wong DJ Tsai MC Hung T Argani P Rinn JL Wang Y Brzoska P Kong B Li R West RB van de Vijver MJ Sukumar S Chang HY Long non-coding RNA HOTAIR reprograms chromatin state to promote cancer metastasis Nature 2010 464 1071 1076 20393566 10.1038/nature08975 PMC3049919

[B9] Prensner JR Iyer MK Balbin OA Dhanasekaran SM Cao Q Brenner JC Laxman B Asangani IA Grasso CS Kominsky HD Cao X Jing X Wang X Siddiqui J Wei JT Robinson D Iyer HK Palanisamy N Maher CA Chinnaiyan AM Transcriptome sequencing across a prostate cancer cohort identifies PCAT-1, an unannotated lincRNA implicated in disease progression Nat Biotechnol 2011 29 742 749 21804560 10.1038/nbt.1914 PMC3152676

[B10] Yap KL Li S Munoz-Cabello AM Raguz S Zeng L Mujtaba S Gil J Walsh MJ Zhou MM Molecular interplay of the noncoding RNA ANRIL and methylated histone H3 lysine 27 by polycomb CBX7 in transcriptional silencing of INK4a Mol Cell 2010 38 662 674 20541999 10.1016/j.molcel.2010.03.021 PMC2886305

[B11] Yoon JH Abdelmohsen K Gorospe M Posttranscriptional gene regulation by long noncoding RNA J Mol Biol 2012 **pii:**S0022-2836(12)00896-0 10.1016/j.jmb.2012.11.024 PMC3594629 23178169

[B12] Mattick JS Deconstructing the dogma: a new view of the evolution and genetic programming of complex organisms Ann N Y Acad Sci 2009 1178 29 46 19845626 10.1111/j.1749-6632.2009.04991.x

[B13] Mattick JS Taft RJ Faulkner GJ A global view of genomic information–moving beyond the gene and the master regulator Trends Genet 2010 26 21 28 19944475 10.1016/j.tig.2009.11.002

[B14] Peterlin BM Brogie JE Price DH 7SK snRNA: a noncoding RNA that plays a major role in regulating eukaryotic transcription Wiley Interdiscip Rev RNA 2012 3 92 103 21853533 10.1002/wrna.106 PMC3223291

[B15] Espinoza CA Allen TA Hieb AR Kugel JF Goodrich JA B2 RNA binds directly to RNA polymerase II to repress transcript synthesis Nat Struct Mol Biol 2004 11 822 829 15300239 10.1038/nsmb812

[B16] Espinoza CA Goodrich JA Kugel JF Characterization of the structure, function, and mechanism of B2 RNA, an ncRNA repressor of RNA polymerase II transcription RNA 2007 13 583 596 17307818 10.1261/rna.310307 PMC1831867

[B17] Yakovchuk P Goodrich JA Kugel JF B2 RNA represses TFIIH phosphorylation of RNA polymerase II Transcription 2011 2 45 49 21326911 10.4161/trns.2.1.14306 PMC3023648

[B18] Sleutels F Zwart R Barlow DP The non-coding Air RNA is required for silencing autosomal imprinted genes Nature 2002 415 810 813 11845212 10.1038/415810a

[B19] Mancini-Dinardo D Steele SJ Levorse JM Ingram RS Tilghman SM Elongation of the Kcnq1ot1 transcript is required for genomic imprinting of neighboring genes Genes Dev 2006 20 1268 1282 16702402 10.1101/gad.1416906 PMC1472902

[B20] Williamson CM Ball ST Dawson C Mehta S Beechey CV Fray M Teboul L Dear TN Kelsey G Peters J Uncoupling antisense-mediated silencing and DNA methylation in the imprinted Gnas cluster PLoS Genet 2011 7 e1001347 21455290 10.1371/journal.pgen.1001347 PMC3063750

[B21] Rinn JL Kertesz M Wang JK Squazzo SL Xu X Brugmann SA Goodnough LH Helms JA Farnham PJ Segal E Chang HY Functional demarcation of active and silent chromatin domains in human HOX loci by noncoding RNAs Cell 2007 129 1311 1323 17604720 10.1016/j.cell.2007.05.022 PMC2084369

[B22] Ng SY Johnson R Stanton LW Human long non-coding RNAs promote pluripotency and neuronal differentiation by association with chromatin modifiers and transcription factors EMBO J 2011 31 522 533 22193719 10.1038/emboj.2011.459 PMC3273385

[B23] Guttman M Rinn JL Modular regulatory principles of large non-coding RNAs Nature 2012 482 339 346 22337053 10.1038/nature10887 PMC4197003

[B24] Wutz A Gene silencing in X-chromosome inactivation: advances in understanding facultative heterochromatin formation Nat Rev Genet 2011 12 542 553 21765457 10.1038/nrg3035

[B25] Ørom UA Derrien T Beringer M Gumireddy K Gardini A Bussotti G Lai F Zytnicki M Notredame C Huang Q Guigo R Shiekhattar R Long noncoding RNAs with enhancer-like function in human cells Cell 2010 143 46 58 20887892 10.1016/j.cell.2010.09.001 PMC4108080

[B26] Wang KC Yang YW Liu B Sanyal A Corces-Zimmerman R Chen Y Lajoie BR Protacio A Flynn RA Gupta RA Wysocka J Lei M Dekker J Helms JA Chang HY A long noncoding RNA maintains active chromatin to coordinate homeotic gene expression Nature 2011 472 120 124 21423168 10.1038/nature09819 PMC3670758

[B27] Magistri M Faghihi MA St Laurent G 3rd Wahlestedt C Regulation of chromatin structure by long noncoding RNAs: focus on natural antisense transcripts Trends Genet 2012 28 389 396 22541732 10.1016/j.tig.2012.03.013 PMC3768148

[B28] Heo JB Sung S Vernalization-mediated epigenetic silencing by a long intronic noncoding RNA Science 2011 331 76 79 21127216 10.1126/science.1197349

[B29] Shearwin KE Callen BP Egan JB Transcriptional interference–a crash course Trends Genet 2005 21 339 345 15922833 10.1016/j.tig.2005.04.009 PMC2941638

[B30] Palmer AC Egan JB Shearwin KE Transcriptional interference by RNA polymerase pausing and dislodgement of transcription factors Transcription 2011 2 9 14 21326903 10.4161/trns.2.1.13511 PMC3023640

[B31] Bird AJ Gordon M Eide DJ Winge DR Repression of ADH1 and ADH3 during zinc deficiency by Zap1-induced intergenic RNA transcripts EMBO J 2006 25 5726 5734 17139254 10.1038/sj.emboj.7601453 PMC1698899

[B32] Bumgarner SL Dowell RD Grisafi P Gifford DK Fink GR Toggle involving cis-interfering noncoding RNAs controls variegated gene expression in yeast Proc Natl Acad Sci U S A 2009 106 18321 18326 19805129 10.1073/pnas.0909641106 PMC2775344

[B33] Petruk S Sedkov Y Riley KM Hodgson J Schweisguth F Hirose S Jaynes JB Brock HW Mazo A Transcription of bxd noncoding RNAs promoted by trithorax represses Ubx in cis by transcriptional interference Cell 2006 127 1209 1221 17174895 10.1016/j.cell.2006.10.039 PMC1866366

[B34] Gummalla M Maeda RK Castro Alvarez JJ Gyurkovics H Singari S Edwards KA Karch F Bender W abd-A regulation by the iab-8 noncoding RNA PLoS Genet 2012 8 e1002720 22654672 10.1371/journal.pgen.1002720 PMC3359974

[B35] Latos PA Pauler FM Koerner MV Şenergin HB Hudson QJ Stocsits RR Allhoff W Stricker SH Klement RM Warczok KE Aumayr K Pasierbek P Barlow DP Airn transcriptional overlap, but not its lncRNA products, induces imprinted Igf2r silencing Science 2012 338 1469 1472 23239737 10.1126/science.1228110

[B36] MacIsaac JL Bogutz AB Morrissy AS Lefebvre L Tissue-specific alternative polyadenylation at the imprinted gene Mest regulates allelic usage at Copg2 Nucleic Acids Res 2012 40 1523 1535 22053079 10.1093/nar/gkr871 PMC3287194

[B37] Wood AJ Schulz R Woodfine K Koltowska K Beechey CV Peters J Bourc'his D Oakey RJ Regulation of alternative polyadenylation by genomic imprinting Genes Dev 2008 22 1141 1146 18451104 10.1101/gad.473408 PMC2335310

[B38] Hobson DJ Wei W Steinmetz LM Svejstrup JQ RNA polymerase II collision interrupts convergent transcription Mol Cell 2012 48 365 374 23041286 10.1016/j.molcel.2012.08.027 PMC3504299

[B39] Kornberg RD Lorch Y Twenty-five years of the nucleosome, fundamental particle of the eukaryote chromosome Cell 1999 98 285 294 10458604 10.1016/s0092-8674(00)81958-3

[B40] Li B Carey M Workman JL The role of chromatin during transcription Cell 2007 128 707 719 17320508 10.1016/j.cell.2007.01.015

[B41] Weiner A Hughes A Yassour M Rando OJ Friedman N High-resolution nucleosome mapping reveals transcription-dependent promoter packaging Genome Res 2010 20 90 100 19846608 10.1101/gr.098509.109 PMC2798834

[B42] Hughes AL Jin Y Rando OJ Struhl K A functional evolutionary approach to identify determinants of nucleosome positioning: a unifying model for establishing the genome-wide pattern Mol Cell 2012 48 5 15 22885008 10.1016/j.molcel.2012.07.003 PMC3472102

[B43] Valouev A Johnson SM Boyd SD Smith CL Fire AZ Sidow A Determinants of nucleosome organization in primary human cells Nature 2011 474 516 520 21602827 10.1038/nature10002 PMC3212987

[B44] Segal E Widom J What controls nucleosome positions? Trends Genet 2009 25 335 343 19596482 10.1016/j.tig.2009.06.002 PMC2810357

[B45] Radman-Livaja M Rando OJ Nucleosome positioning: how is it established, and why does it matter? Dev Biol 2010 339 258 266 19527704 10.1016/j.ydbio.2009.06.012 PMC2830277

[B46] Martens JA Laprade L Winston F Intergenic transcription is required to repress the Saccharomyces cerevisiae SER3 gene Nature 2004 429 571 574 15175754 10.1038/nature02538

[B47] Belotserkovskaya R Oh S Bondarenko VA Orphanides G Studitsky VM Reinberg D FACT facilitates transcription-dependent nucleosome alteration Science 2003 301 1090 1093 12934006 10.1126/science.1085703

[B48] Reinberg D Sims RJ 3rd de FACTo nucleosome dynamics J Biol Chem 2006 281 23297 23301 16766522 10.1074/jbc.R600007200

[B49] Nourani A Robert F Winston F Evidence that Spt2/Sin1, an HMG-like factor, plays roles in transcription elongation, chromatin structure, and genome stability in Saccharomyces cerevisiae Mol Cell Biol 2006 26 1496 1509 16449659 10.1128/MCB.26.4.1496-1509.2006 PMC1367203

[B50] Hainer SJ Pruneski JA Mitchell RD Monteverde RM Martens JA Intergenic transcription causes repression by directing nucleosome assembly Genes Dev 2011 25 29 40 21156811 10.1101/gad.1975011 PMC3012934

[B51] Thebault P Boutin G Bhat W Rufiange A Martens J Nourani A Transcription regulation by the noncoding RNA SRG1 requires Spt2-dependent chromatin deposition in the wake of RNA polymerase II Mol Cell Biol 2011 31 1288 1300 21220514 10.1128/MCB.01083-10 PMC3067896

[B52] Kaplan CD Laprade L Winston F Transcription elongation factors repress transcription initiation from cryptic sites Science 2003 301 1096 1099 12934008 10.1126/science.1087374

[B53] Cheung V Chua G Batada NN Landry CR Michnick SW Hughes TR Winston F Chromatin- and transcription-related factors repress transcription from within coding regions throughout the Saccharomyces cerevisiae genome PLoS Biol 2008 6 e277 18998772 10.1371/journal.pbio.0060277 PMC2581627

[B54] Gallastegui E Millan-Zambrano G Terme JM Chavez S Jordan A Chromatin reassembly factors are involved in transcriptional interference promoting HIV latency J Virol 2011 85 3187 3202 21270164 10.1128/JVI.01920-10 PMC3067836

[B55] Bannister AJ Kouzarides T Regulation of chromatin by histone modifications Cell Res 2011 21 381 395 21321607 10.1038/cr.2011.22 PMC3193420

[B56] Brookes E Pombo A Modifications of RNA polymerase II are pivotal in regulating gene expression states EMBO Rep 2009 10 1213 1219 19834511 10.1038/embor.2009.221 PMC2775184

[B57] Ehrensberger AH Svejstrup JQ Reprogramming chromatin Crit Rev Biochem Mol Biol 2012 47 464 482 22757592 10.3109/10409238.2012.697125

[B58] Ng HH Robert F Young RA Struhl K Targeted recruitment of Set1 histone methylase by elongating Pol II provides a localized mark and memory of recent transcriptional activity Mol Cell 2003 11 709 719 12667453 10.1016/s1097-2765(03)00092-3

[B59] Krogan NJ Kim M Tong A Golshani A Cagney G Canadien V Richards DP Beattie BK Emili A Boone C Shilatifard A Buratowski S Greenblatt J Methylation of histone H3 by Set2 in Saccharomyces cerevisiae is linked to transcriptional elongation by RNA polymerase II Mol Cell Biol 2003 23 4207 4218 12773564 10.1128/MCB.23.12.4207-4218.2003 PMC427527

[B60] Schneider R Bannister AJ Myers FA Thorne AW Crane-Robinson C Kouzarides T Histone H3 lysine 4 methylation patterns in higher eukaryotic genes Nat Cell Biol 2004 6 73 77 14661024 10.1038/ncb1076

[B61] Carrozza MJ Li B Florens L Suganuma T Swanson SK Lee KK Shia WJ Anderson S Yates J Washburn MP Workman JL Histone H3 methylation by Set2 directs deacetylation of coding regions by Rpd3S to suppress spurious intragenic transcription Cell 2005 123 581 592 16286007 10.1016/j.cell.2005.10.023

[B62] Kim T Buratowski S Dimethylation of H3K4 by Set1 recruits the Set3 histone deacetylase complex to 5' transcribed regions Cell 2009 137 259 272 19379692 10.1016/j.cell.2009.02.045 PMC2802783

[B63] Keogh MC Kurdistani SK Morris SA Ahn SH Podolny V Collins SR Schuldiner M Chin K Punna T Thompson NJ Boone C Emili A Weissman JS Hughes TR Strahl BD Grunstein M Greenblatt JF Buratowski S Krogan NJ Cotranscriptional set2 methylation of histone H3 lysine 36 recruits a repressive Rpd3 complex Cell 2005 123 593 605 16286008 10.1016/j.cell.2005.10.025

[B64] van Werven FJ Neuert G Hendrick N Lardenois A Buratowski S van Oudenaarden A Primig M Amon A Transcription of two long noncoding RNAs mediates mating-type control of gametogenesis in budding yeast Cell 2012 150 1170 1181 22959267 10.1016/j.cell.2012.06.049 PMC3472370

[B65] Houseley J Rubbi L Grunstein M Tollervey D Vogelauer M A ncRNA modulates histone modification and mRNA induction in the yeast GAL gene cluster Mol Cell 2008 32 685 695 19061643 10.1016/j.molcel.2008.09.027 PMC7610895

[B66] Kim T Xu Z Clauder-Munster S Steinmetz LM Buratowski S Set3 HDAC mediates effects of overlapping noncoding transcription on gene induction kinetics Cell 2012 150 1158 1169 22959268 10.1016/j.cell.2012.08.016 PMC3461055

[B67] Jones PA Functions of DNA methylation: islands, start sites, gene bodies and beyond Nat Rev Genet 2012 13 484 492 22641018 10.1038/nrg3230

[B68] Deaton AM Bird A CpG islands and the regulation of transcription Genes Dev 2011 25 1010 1022 21576262 10.1101/gad.2037511 PMC3093116

[B69] Ooi SK O'Donnell AH Bestor TH Mammalian cytosine methylation at a glance J Cell Sci 2009 122 2787 2791 19657014 10.1242/jcs.015123 PMC2724605

[B70] Ooi SK Qiu C Bernstein E Li K Jia D Yang Z Erdjument-Bromage H Tempst P Lin SP Allis CD Cheng X Bestor TH DNMT3L connects unmethylated lysine 4 of histone H3 to de novo methylation of DNA Nature 2007 448 714 717 17687327 10.1038/nature05987 PMC2650820

[B71] Tufarelli C Stanley JA Garrick D Sharpe JA Ayyub H Wood WG Higgs DR Transcription of antisense RNA leading to gene silencing and methylation as a novel cause of human genetic disease Nat Genet 2003 34 157 165 12730694 10.1038/ng1157

[B72] Ligtenberg MJ Kuiper RP Chan TL Goossens M Hebeda KM Voorendt M Lee TY Bodmer D Hoenselaar E Hendriks-Cornelissen SJ Tsui WY Kong CK Brunner HG van Kessel AG Yuen ST van Krieken JH Leung SY Hoogerbrugge N Heritable somatic methylation and inactivation of MSH2 in families with Lynch syndrome due to deletion of the 3' exons of TACSTD1 Nat Genet 2009 41 112 117 19098912 10.1038/ng.283

[B73] Santoro F Barlow DP Developmental control of imprinted expression by macro non-coding RNAs Semin Cell Dev Biol 2011 22 328 335 21333747 10.1016/j.semcdb.2011.02.018

[B74] Santoro F Mayer D Klement RM Warczok KE Stukalov A Barlow DP Pauler FM Imprinted Igf2r silencing depends on continuous Airn lncRNA expression and is not restricted to a developmental window Development 2013 140 1184 1195 23444351 10.1242/dev.088849

[B75] Pauler FM Barlow DP Hudson QJ Mechanisms of long range silencing by imprinted macro non-coding RNAs Curr Opin Genet Dev 2012 22 283 289 22386265 10.1016/j.gde.2012.02.005 PMC3387373

[B76] Pauler FM Koerner MV Barlow DP Silencing by imprinted noncoding RNAs: is transcription the answer? Trends Genet 2007 23 284 292 17445943 10.1016/j.tig.2007.03.018 PMC2847181

[B77] Barlow DP Genomic imprinting: a mammalian epigenetic discovery model Annu Rev Genet 2011 45 379 403 21942369 10.1146/annurev-genet-110410-132459

[B78] Koerner MV Pauler FM Huang R Barlow DP The function of non-coding RNAs in genomic imprinting Development 2009 136 1771 1783 19429783 10.1242/dev.030403 PMC2847617

[B79] Wang ZQ Fung MR Barlow DP Wagner EF Regulation of embryonic growth and lysosomal targeting by the imprinted Igf2/Mpr gene Nature 1994 372 464 467 7984240 10.1038/372464a0

[B80] Pauler FM Stricker SH Warczok KE Barlow DP Long-range DNase I hypersensitivity mapping reveals the imprinted Igf2r and Air promoters share cis-regulatory elements Genome Res 2005 15 1379 1387 16204191 10.1101/gr.3783805 PMC1240080

[B81] Stoger R Kubicka P Liu CG Kafri T Razin A Cedar H Barlow DP Maternal-specific methylation of the imprinted mouse Igf2r locus identifies the expressed locus as carrying the imprinting signal Cell 1993 73 61 71 8462104 10.1016/0092-8674(93)90160-r

[B82] Racanelli AC Turner FB Xie LY Taylor SM Moran RG A mouse gene that coordinates epigenetic controls and transcriptional interference to achieve tissue-specific expression Mol Cell Biol 2008 28 836 848 17998333 10.1128/MCB.01088-07 PMC2223435

[B83] Palmer AC Ahlgren-Berg A Egan JB Dodd IB Shearwin KE Potent transcriptional interference by pausing of RNA polymerases over a downstream promoter Mol Cell 2009 34 545 555 19524535 10.1016/j.molcel.2009.04.018 PMC2697128

[B84] Adelman K Lis JT Promoter-proximal pausing of RNA polymerase II: emerging roles in metazoans Nat Rev Genet 2012 13 720 731 22986266 10.1038/nrg3293 PMC3552498

[B85] Levine M Paused RNA polymerase II as a developmental checkpoint Cell 2011 145 502 511 21565610 10.1016/j.cell.2011.04.021 PMC4257488

[B86] Nagano T Mitchell JA Sanz LA Pauler FM Ferguson-Smith AC Feil R Fraser P The Air noncoding RNA epigenetically silences transcription by targeting G9a to chromatin Science 2008 322 1717 1720 18988810 10.1126/science.1163802

[B87] Mager J Montgomery ND de Villena FP Magnuson T Genome imprinting regulated by the mouse Polycomb group protein Eed Nat Genet 2003 33 502 507 12627233 10.1038/ng1125

[B88] Terranova R Yokobayashi S Stadler MB Otte AP van Lohuizen M Orkin SH Peters AH Polycomb group proteins Ezh2 and Rnf2 direct genomic contraction and imprinted repression in early mouse embryos Dev Cell 2008 15 668 679 18848501 10.1016/j.devcel.2008.08.015

[B89] Korostowski L Sedlak N Engel N The Kcnq1ot1 long non-coding RNA affects chromatin conformation and expression of Kcnq1, but does not regulate its imprinting in the developing heart PLoS Genet 2012 8 e1002956 23028363 10.1371/journal.pgen.1002956 PMC3447949

[B90] Visel A Rubin EM Pennacchio LA Genomic views of distant-acting enhancers Nature 2009 461 199 205 19741700 10.1038/nature08451 PMC2923221

[B91] Ong CT Corces VG Enhancers: emerging roles in cell fate specification EMBO Rep 2012 13 423 430 22491032 10.1038/embor.2012.52 PMC3343362

[B92] Kim TK Hemberg M Gray JM Costa AM Bear DM Wu J Harmin DA Laptewicz M Barbara-Haley K Kuersten S Markenscoff-Papadimitriou E Kuhl D Bito H Worley PF Kreiman G Greenberg ME Widespread transcription at neuronal activity-regulated enhancers Nature 2010 465 182 187 20393465 10.1038/nature09033 PMC3020079

[B93] Schatz DG Swanson PC V(D)J recombination: mechanisms of initiation Annu Rev Genet 2011 45 167 202 21854230 10.1146/annurev-genet-110410-132552

[B94] Bolland DJ Wood AL Afshar R Featherstone K Oltz EM Corcoran AE Antisense intergenic transcription precedes Igh D-to-J recombination and is controlled by the intronic enhancer Emu Mol Cell Biol 2007 27 5523 5533 17526723 10.1128/MCB.02407-06 PMC1952079

[B95] Giallourakis CC Franklin A Guo C Cheng HL Yoon HS Gallagher M Perlot T Andzelm M Murphy AJ Macdonald LE Yancopoulos GD Alt FW Elements between the IgH variable (V) and diversity (D) clusters influence antisense transcription and lineage-specific V(D)J recombination Proc Natl Acad Sci U S A 2010 107 22207 22212 21123744 10.1073/pnas.1015954107 PMC3009784

[B96] Abarrategui I Krangel MS Noncoding transcription controls downstream promoters to regulate T-cell receptor alpha recombination EMBO J 2007 26 4380 4390 17882258 10.1038/sj.emboj.7601866 PMC2034674

[B97] Ashe HL Monks J Wijgerde M Fraser P Proudfoot NJ Intergenic transcription and transinduction of the human beta-globin locus Genes Dev 1997 11 2494 2509 9334315 10.1101/gad.11.19.2494 PMC316561

[B98] Gribnau J Diderich K Pruzina S Calzolari R Fraser P Intergenic transcription and developmental remodeling of chromatin subdomains in the human beta-globin locus Mol Cell 2000 5 377 386 10882078 10.1016/s1097-2765(00)80432-3

[B99] Cumberledge S Zaratzian A Sakonju S Characterization of two RNAs transcribed from the cis-regulatory region of the abd-A domain within the Drosophila bithorax complex Proc Natl Acad Sci U S A 1990 87 3259 3263 1692133 10.1073/pnas.87.9.3259 PMC53879

[B100] Beisel C Paro R Silencing chromatin: comparing modes and mechanisms Nat Rev Genet 2011 12 123 135 21221116 10.1038/nrg2932

[B101] Schmitt S Prestel M Paro R Intergenic transcription through a polycomb group response element counteracts silencing Genes Dev 2005 19 697 708 15741315 10.1101/gad.326205 PMC1065723

[B102] Sessa L Breiling A Lavorgna G Silvestri L Casari G Orlando V Noncoding RNA synthesis and loss of Polycomb group repression accompanies the colinear activation of the human HOXA cluster RNA 2007 13 223 239 17185360 10.1261/rna.266707 PMC1781374

[B103] Kanhere A Viiri K Araújo CC Rasaiyaah J Bouwman RD Whyte WA Pereira CF Brookes E Walker K Bell GW Pombo A Fisher AG Young RA Jenner RG Short RNAs are transcribed from repressed polycomb target genes and interact with polycomb repressive complex-2 Mol Cell 2010 38 675 688 20542000 10.1016/j.molcel.2010.03.019 PMC2886029

[B104] Hekimoglu-Balkan B Aszodi A Heinen R Jaritz M Ringrose L Intergenic Polycomb target sites are dynamically marked by non-coding transcription during lineage commitment RNA Biol 9 314 325 22336714 10.4161/rna.19102 PMC3384584

[B105] Uhler JP Hertel C Svejstrup JQ A role for noncoding transcription in activation of the yeast PHO5 gene Proc Natl Acad Sci U S A 2007 104 8011 8016 17470801 10.1073/pnas.0702431104 PMC1859995

[B106] Neil H Malabat C d'Aubenton-Carafa Y Xu Z Steinmetz LM Jacquier A Widespread bidirectional promoters are the major source of cryptic transcripts in yeast Nature 2009 457 1038 1042 19169244 10.1038/nature07747

[B107] Seila AC Calabrese JM Levine SS Yeo GW Rahl PB Flynn RA Young RA Sharp PA Divergent transcription from active promoters Science 2008 322 1849 1851 19056940 10.1126/science.1162253 PMC2692996

[B108] Brosius J Waste not, want not–transcript excess in multicellular eukaryotes Trends Genet 2005 21 287 288 15851065 10.1016/j.tig.2005.02.014

[B109] Kowalczyk MS Higgs DR Gingeras TR Molecular biology: RNA discrimination Nature 2012 482 310 311 22337043 10.1038/482310a

[B110] Wang GZ Lercher MJ Hurst LD Transcriptional coupling of neighboring genes and gene expression noise: evidence that gene orientation and noncoding transcripts are modulators of noise Genome Biol Evol 2011 3 320 331 21402863 10.1093/gbe/evr025 PMC5654408

[B111] Ulitsky I Shkumatava A Jan CH Sive H Bartel DP Conserved function of lincRNAs in vertebrate embryonic development despite rapid sequence evolution Cell 2011 147 1537 1550 22196729 10.1016/j.cell.2011.11.055 PMC3376356

[B112] Zeng Y Cullen BR RNA interference in human cells is restricted to the cytoplasm RNA 2002 8 855 860 12166640 10.1017/s1355838202020071 PMC1370302

[B113] Ohrt T Muetze J Svoboda P Schwille P Intracellular localization and routing of miRNA and RNAi pathway components Curr Top Med Chem 2012 12 79 88 22196276 10.2174/156802612798919132

[B114] Ideue T Hino K Kitao S Yokoi T Hirose T Efficient oligonucleotide-mediated degradation of nuclear noncoding RNAs in mammalian cultured cells RNA 2009 15 1578 1587 19535462 10.1261/rna.1657609 PMC2714749

[B115] Tse MT Antisense therapeutics: Nuclear RNA more susceptible to knockdown Nat Rev Drug Discov 2012 11 674 22903045 10.1038/nrd3825

[B116] Zhang B Arun G Mao YS Lazar Z Hung G Bhattacharjee G Xiao X Booth CJ Wu J Zhang C Spector DL The lncRNA Malat1 is dispensable for mouse development but its transcription plays a cis-regulatory role in the adult Cell Rep 2012 2 111 123 22840402 10.1016/j.celrep.2012.06.003 PMC3408587

[B117] Nakagawa S Naganuma T Shioi G Hirose T Paraspeckles are subpopulation-specific nuclear bodies that are not essential in mice J Cell Biol 193 31 39 21444682 10.1083/jcb.201011110 PMC3082198

[B118] Eißmann M Gutschner T Hämmerle M Günther S Caudron-Herger M Groß M Schirmacher P Rippe K Braun T Zörnig M Diederichs S Loss of the abundant nuclear non-coding RNA MALAT1 is compatible with life and development RNA Biol 2012 9 1076 1087 22858678 10.4161/rna.21089 PMC3551862

[B119] Nakagawa S Ip JY Shioi G Tripathi V Zong X Hirose T Prasanth KV Malat1 is not an essential component of nuclear speckles in mice RNA 2012 18 1487 1499 22718948 10.1261/rna.033217.112 PMC3404370

[B120] Schorderet P Duboule D Structural and functional differences in the long non-coding RNA hotair in mouse and human PLoS Genet 2011 7 e1002071 21637793 10.1371/journal.pgen.1002071 PMC3102750

[B121] Guenzl PM Barlow DP Macro lncRNAs: A new layer of cis-regulatory information in the mammalian genome RNA Biol 2012 9 731 741 22617879 10.4161/rna.19985

[B122] Carette JE Guimaraes CP Wuethrich I Blomen VA Varadarajan M Sun C Bell G Yuan B Muellner MK Nijman SM Ploegh HL Brummelkamp TR Global gene disruption in human cells to assign genes to phenotypes by deep sequencing Nat Biotechnol 2011 29 542 546 21623355 10.1038/nbt.1857 PMC3111863

[B123] Wirt SE Porteus MH Development of nuclease-mediated site-specific genome modification Curr Opin Immunol 2012 24 609 616 22981684 10.1016/j.coi.2012.08.005

[B124] Mali P Yang L Esvelt KM Aach J Guell M Dicarlo JE Norville JE Church GM RNA-Guided Human Genome Engineering via Cas9 Science 2013 339 823 826 23287722 10.1126/science.1232033 PMC3712628

[B125] Latos PA Stricker SH Steenpass L Pauler FM Huang R Senergin BH Regha K Koerner MV Warczok KE Unger C Barlow DP An in vitro ES cell imprinting model shows that imprinted expression of the Igf2r gene arises from an allele-specific expression bias Development 2009 136 437 448 19141673 10.1242/dev.032060 PMC2846269

[B126] Kohama C Kato H Numata K Hirose M Takemasa T Ogura A Kiyosawa H ES cell differentiation system recapitulates the establishment of imprinted gene expression in a cell-type-specific manner Hum Mol Genet 2012 21 1391 1401 22156770 10.1093/hmg/ddr577

[B127] Rosenbloom KR Sloan CA Malladi VS Dreszer TR Learned K Kirkup VM Wong MC Maddren M Fang R Heitner SG Lee BT Barber GP Harte RA Diekhans M Long JC Wilder SP Zweig AS Karolchik D Kuhn RM Haussler D Kent WJ ENCODE Data in the UCSC Genome Browser: year 5 update Nucleic Acids Res 2013 41 D56 63 23193274 10.1093/nar/gks1172 PMC3531152

[B128] Loven J Orlando DA Sigova AA Lin CY Rahl PB Burge CB Levens DL Lee TI Young RA Revisiting global gene expression analysis Cell 2012 151 476 482 23101621 10.1016/j.cell.2012.10.012 PMC3505597

[B129] Clark MB Mattick JS Long noncoding RNAs in cell biology Semin Cell Dev Biol 2011 22 366 376 21256239 10.1016/j.semcdb.2011.01.001

